# Impact of clinical response and treatment tolerability on HRQoL in newly diagnosed Philadelphia chromosome-positive acute lymphoblastic leukemia patients treated with ponatinib or imatinib

**DOI:** 10.1007/s00277-025-06635-0

**Published:** 2025-10-10

**Authors:** Ajibade Ashaye, Ling Shi, Ibrahim Aldoss, Pau Montesinos, Pankit Vachhani, Vanderson Rocha, Cristina Papayannidis, Jessica T. Leonard, Maria R. Baer, Jose-Maria Ribera, Yanyu Wu, Meliessa Hennessy, Alexandar Vorog, Shien Guo

**Affiliations:** 1 Takeda Development Center Americas, Inc, Cambridge, MA USA; 2 Evidera, PPD clinical research business of Thermo Fisher Scientific, Waltham, MA USA; 3 City of Hope National Medical Center, Duarte, CA USA; 4 Hospital Universitari i Politècnic La Fe, Valencia, Spain; 5 University of Alabama at Birmingham, Birmingham, AL USA; 6 Instituto do Câncer do Estado de São Paulo, University of São Paulo, São Paulo, Brazil; 7 IRCCS Azienda Ospedaliero-Universitaria di Bologna, Istituto di Ematologia “L. e A. Seràgnoli,” Bologna, Bologna, Italy; 8 Oregon Health and Science University, Portland, OR USA; 9 University of Maryland Marlene and Stewart Greenebaum Comprehensive Cancer Center, Baltimore, MD USA; 10 ICO – Hospital Germans Trias i Pujol, Josep Carreras Leukaemia Research Institute, Badalona, Spain

**Keywords:** Acute lymphoblastic leukemia, Clinical response, Health-related quality of life, Imatinib, Ponatinib, Treatment tolerability

## Abstract

In the phase 3 PhALLCON trial (NCT03589326), ponatinib demonstrated superior efficacy, patient-reported treatment tolerability, and health-related quality of life (HRQoL) compared to imatinib in adults with newly diagnosed Philadelphia chromosome-positive (Ph+) acute lymphoblastic leukemia (ALL). To explore the association between clinical response and HRQoL and substantiate the superior effect of ponatinib over imatinib on HRQoL, we analyzed the impact of clinical response and treatment tolerability on changes in HRQoL.

HRQoL was assessed using the Functional Assessment of Cancer Therapy–Leukemia (FACT-Leu) questionnaire and the EQ-5D-5 L. Treatment tolerability was assessed using the FACT-GP5 item “bothered by treatment side effects.” Linear mixed-effects regression models were used to examine changes in HRQoL over time, with clinical response status and patient-reported overall treatment tolerability as time-varying predictors, while controlling for significant covariates.

This analysis included data from 238 patients (159 ponatinib, 79 imatinib). Achieving clinical response (complete remission or incomplete remission) was associated with significantly better changes from baseline across all FACT-Leu domains and the EQ-visual analogue scale than not achieving clinical response (*p* < 0.05). Treatment-related side effects led to significantly and meaningfully worse changes in HRQoL than “not bothered by treatment,” with higher levels of “bother” associated with greater worsening in HRQoL from baseline.

Taken together with the better treatment tolerability and longer response duration of ponatinib compared to imatinib, these findings further substantiate the HRQoL benefit of ponatinib over imatinib in patients with Ph + ALL.

**Supplementary Information:**

The online version contains supplementary material available at 10.1007/s00277-025-06635-0.

## Introduction

Acute lymphoblastic leukemia (ALL) is a malignant hematologic disorder characterized by proliferation of immature lymphoid cells (lymphoblasts) [1]. Approximately 25% of adults with ALL have the Philadelphia chromosome (Ph + ALL) [2, 3]. Standard-of-care frontline therapy for Ph + ALL includes treatment with a BCR::ABL1 tyrosine kinase inhibitor (TKI), combined with chemotherapy [4, 5]. Treatment with a first-generation (imatinib) or second-generation (dasatinib, nilotinib) TKI has significantly improved response rates and survival outcomes in patients with Ph + ALL [6]. However, BCR::ABL1 mutations such as T315I confer resistance to treatment with first- and second-generation TKIs [7, 8].

Ponatinib is a third-generation TKI that can effectively inhibit BCR::ABL1 with or without any single-mutation variants, including T315I [9, 10]. In the phase 3 PhALLCON trial, ponatinib, compared to imatinib, in combination with reduced-intensity chemotherapy produced a significantly higher rate of minimal residual disease (MRD)-negative complete remission (CR; 34% vs. 17%; *p* = 0.002) at the end of induction in adults with newly diagnosed Ph + ALL [6]. While the overall response rate based on cumulative CR or CR with incomplete hematologic recovery (CRi) by the end of induction was comparable between treatment groups (94% for ponatinib vs. 98% for imatinib), duration of response from CR to relapse was longer for ponatinib (median time not reached) than for imatinib (22.3 months) [6]. Based on the PhALLCON trial data, the US Food and Drug Administration (FDA) granted accelerated approval for ponatinib with chemotherapy for treatment of adults with newly diagnosed Ph + ALL [11, 12]. Additionally, patient-reported outcome (PRO) data from the PhALLCON trial indicated that ponatinib had superior overall treatment tolerability, as assessed by the Functional Assessment of Cancer Therapy (FACT) Item-GP5 [13]. 38% of PhALLCON trial patients reported “no bother” from side effects at the end of treatment with ponatinib, compared to 18% of patients treated with imatinib (*p* = 0.017) [13]. Patients treated with ponatinib also reported a greater improvement in HRQoL, as assessed by the FACT-Leukemia (FACT-Leu) and EQ-5D-5 L, at the end of induction and consolidation phases compared to patients treated with imatinib [13].

PRO data provide invaluable insights into patients’ perceptions regarding the impact of cancer and its treatment on their health-related quality of life (HRQoL), especially given the significant toxicity associated with cancer therapies [14]. PRO data are particularly important when the clinical benefits of new cancer treatments are evaluated for accelerated approval based on the effect of a surrogate endpoint (e.g., rate of CR or event/progression-free survival), which is considered reasonably likely to predict clinical benefit in serious or life-threatening diseases but is itself not a direct measure of clinical benefit [14, 15]. These surrogate endpoints are generally less well-established than those for traditional approvals, which are known to predict clinical benefit (e.g., blood pressure in cardiovascular disease) [16]. Further, since overall survival data for new cancer treatments are often immature at the time of regulatory approval, PRO data may provide evidence regarding the benefits of a treatment in the absence of mature overall survival data.

This study evaluated the impact of clinical response and patient-reported overall treatment tolerability on HRQoL in PhALLCON trial patients treated with ponatinib or imatinib in combination with reduced-intensity chemotherapy. The objective of this study was to explore the association between clinical response (i.e., CR and MRD-negative CR, considered as surrogate endpoints according to the FDA’s definition [15]) and HRQoL, and to provide deeper insights into the HRQoL benefits of ponatinib over imatinib that have been previously reported [13].

## Methods

### Study design

PhALLCON is a phase 3, randomized, international, open-label, multicenter trial evaluating the safety and efficacy of ponatinib vs. imatinib, administered in combination with reduced-intensity chemotherapy as first-line therapy in adults with newly diagnosed Ph + ALL (NCT03589326). Details regarding the study design and conduct of the PhALLCON trial have been reported previously [6].

Briefly, participants were randomized 2:1 to receive an oral dose of ponatinib (30 mg daily) or imatinib (600 mg daily) in combination with 20 cycles of a reduced-intensity chemotherapy regimen (cycles 1–3 of induction therapy with TKI plus vincristine and dexamethasone, cycles 4–9 of consolidation therapy with TKI plus methotrexate and cytarabine, and cycles 10–20 of maintenance therapy with TKI plus vincristine and prednisone), followed by single-agent therapy with ponatinib or imatinib. Participants continued to receive treatment until relapse from CR, disease progression, unacceptable toxicity, withdrawal of consent, receipt of hematopoietic stem cell transplantation or alternative therapy, or death. The primary endpoint was MRD-negative CR at the end of induction. The effect of ponatinib on patient-reported HRQoL and overall treatment tolerability was compared with that of imatinib as an exploratory objective.

The study protocol was approved by the institutional review boards or ethics committees of all participating centers. The trial was conducted in accordance with the International Council for Harmonisation (ICH) Guideline for Good Clinical Practice and the Declaration of Helsinki. All participants provided written informed consent.

### Participants

Participants were adults (age ≥ 18 years) with newly diagnosed Ph + ALL, had an Eastern Cooperative Oncology Group performance status of ≤ 2, and did not have clinically significant or uncontrolled cardiovascular disease. Details regarding the eligibility criteria for the PhALLCON trial have been reported previously [6].

### Clinical response, treatment tolerability, and HRQoL assessments

Clinical response was assessed at the end of cycles (C) 1 and C2, end of induction (C3), and during or at the end of consolidation (end of C9 or end of study treatment, whichever occurred earlier). Clinical response was defined as investigator-reported CR or CRi. Investigator-reported CR was defined as meeting all of the following criteria for at least 4 weeks: no circulating blasts and < 5% blasts in the bone marrow; normal maturation of all cellular components in the bone marrow; no extramedullary disease (including central nervous system involvement, lymphadenopathy, splenomegaly, skin/gum infiltration, testicular mass); absolute neutrophil count ≥ 1.0 × 10^9^/L; and platelet count ≥ 100 × 10^9^/L. CRi was defined as meeting all criteria for CR except platelet count or absolute neutrophil count. Additionally, clinical response was also defined based on depth of molecular response (MR) levels. MRD-negative CR was defined as meeting the criteria for CR at or after the end of induction (~ 3 months) with a BCR-ABL1/ABL1 ratio ≤ 0.01% or undetectable BCR::ABL1 transcripts in complementary DNA with ≥ 10,000 ABL1 transcripts (i.e., MR 4) [6].

Treatment tolerability was assessed by the patient-reported FACT-Item GP5 (‘I am bothered by side effects of treatment’), with response levels ranging from 0 (“Not at all”) to 4 (“Very much”) [17].

HRQoL was assessed using the patient-reported FACT-Leu questionnaire [18] and the EQ-5D-5L [19] at screening/baseline, then C1D1, C4D1, C7D1, C10D1, C13D1, C16D1, C19D1, and C21D1, and at least every 6 cycles thereafter and at the end of treatment. Participants completed electronic and/or paper forms of the PRO questionnaires at the scheduled assessment visits.

The FACT-Leu includes a 27-item FACT-G subscale and a 17-item ‘Additional Concerns’ subscale (FACT-LeuS) that assesses symptoms and concerns related to leukemia **(Table ****S1**) [18, 20]. The FACT-Leu primary domains included in the analyses were selected based on their clinical relevance and importance to patients with Ph + ALL: FACT-G physical well-being (PWB), FACT-G total score, FACT-LeuS, FACT-Leu trial outcome index (TOI), and FACT-Leu total score [21, 22]. The FACT-G PWB domain includes items to assess the impact of disease and treatment on the patient’s physical functioning and quality of life. The FACT-G total score is the sum all FACT-G domain scores (physical, emotional, mental, and social well-being scores). The FACT-LeuS includes leukemia-specific items measuring symptoms such as fever, pain, chills, night sweats, lumps or swelling, bleeding, bruising, fatigue, emotional disturbance, and feeling of isolation. The FACT-Leu TOI is the sum of the FACT-G PWB, FACT-G functional well-being, and FACT-LeuS scores [20]. The FACT-Leu total score is the sum of the FACT-G and FACT-LeuS scores [20].

The EQ-5D-5L is a five-item self-reported measure of functioning and well-being [19]. A weighted health utility index (HUI) was estimated by cross-walking to country-specific EQ-5D-3L value sets (UK and US) [23, 24]. The EQ-5D-3L utility index ranges from − 0.594 to 1.0 based on the UK population and − 0.109 to 1.0 based on the US population. A higher score represents a better health state [23, 24]. The EQ-5D-5L also contains a visual analogue scale (EQ-VAS) ranging from 0 to 100 (0=“worst imaginable health” and 100=“best imaginable health”). The mean change in domain score considered clinically meaningful to patients was based on published minimal important difference (MID) thresholds [25–30].

### Statistical analyses

The data cutoff date was August 12, 2022. Participants in the intent-to-treat population (all patients who were randomly assigned) who had baseline and at least one post-baseline PRO assessment were included in the analyses. All analyses used pooled data from the ponatinib and imatinib arms.

Linear mixed-effects regression models for repeated-measures (MMRM) [31] with random intercept and slope were used to evaluate the associations between key predictors of interest (i.e., investigator-reported clinical response, molecular response, and patient-reported overall treatment tolerability) and changes in HRQoL over time. The MMRM is widely regarded as the standard approach for analyzing longitudinal data with continuous endpoints, as it is efficient and reliable in handling within-subject correlations and missing data [32]. Specifically, it handles missing data using maximum likelihood approach, based on the assumption of missing at random (MAR). It assumes patients with missing data would follow a similar trajectory to other patients with the same covariates at that timepoint [33]. The MMRM model performed for this analysis used an unstructured covariance matrix to obtain the random effects variance components. The change at each of the post-baseline scheduled visits from baseline for each of the selected domains of interest was used as the dependent variable, with clinical response and treatment tolerability as time-varying predictors, and baseline demographic and disease characteristics as covariates (Table S2). For time-varying factors (i.e., clinical response status and treatment tolerability), the value assigned at each PRO assessment visit was based on the corresponding assessment on or before the PRO assessment date. For example, if a patient completed a PRO assessment at C7D1, and clinical response and treatment tolerability were also evaluated at that visit, the data from C7D1 would be used. If the next PRO assessment occurred on C10D1 without a new clinical or tolerability assessment, the values from C7D1 would be carried forward, assuming no change in status had occurred in the interim.

The MMRM analyses were conducted in two stages. In the first stage, baseline factors were analyzed in univariate models that included time (PRO assessment visit, modelled as a discrete variable) and baseline domain score as covariates. In the second stage, baseline factors with a p-value < 0.1 in the univariate analyses were included as covariates in the multivariable models, with clinical response and patient-reported overall treatment tolerability as time-varying predictors, and time (PRO assessment visit) and baseline domain score as covariates. Clinical response was analyzed individually (i.e., CR/CRi vs. no CR/CRi; MRD-negative CR and MRD-positive CR vs. no CR; MR levels defined by BCR-ABL1/ABL1 ratio) due to collinearity among the levels. Overall treatment tolerability was examined in the same models. Non-significant covariates (*p* > 0.05) were removed one at a time so that the final models retained only significant covariates (*p* < 0.05). The interactions between treatment and clinical response, as well as between treatment and overall treatment tolerability, were examined in the final models to assess whether the relationship between these factors differed by treatment group.

Statistical significance was defined as *p* < 0.05. Due to the exploratory nature of this analysis, no adjustment for multiplicity were performed. All statistical analyses were conducted using SAS^®^ version 9.4 (SAS Institute, Cary, NC).

## Results

### Participants

A total of 245 patients (ponatinib 164, imatinib 81) were randomized in the PhALLCON study [6]. Of these, 238 (97%) patients (ponatinib 159, imatinib 79) had at least one PRO assessment and were included in the analysis. Most participants were 45 years or older (64%), females (54%), White (67%), and not Hispanic or Latino (66%) (Table 1). Baseline PRO scores and PRO completion rates at each assessment visit have been previously reported [13]. The PRO completion by clinical response status was summarized in Table S3. Almost all patients achieved CR/CRi, although the time to achieve CR/CRi and duration of response varied among patients.

**Table 1:** Patient demographics and clinical characteristics (*N* = 238)

Characteristics	*n* (%)
Age (years)	
18 to < 45	83 (34.9)
45 to < 60	65 (27.3)
≥ 60	90 (37.8)
Sex	
Female	129 (54.2)
Male	109 (45.8)
Race	
American Indian or Alaska Native	4 (1.7)
Asian	31 (13.0)
Black or African American	12 (5.0)
White	161 (67.6)
Multiple	1 (0.4)
Not reported	29 (12.2)
Ethnicity	
Hispanic or Latino	57 (23.9)
Not Hispanic or Latino	157 (66.0)
Unknown ^a^	24 (10.1)
ECOG performance status	
0	102 (42.9)
1	124 (52.1)
2	12 (5.0)
BCR::ABL transcript type	
e1a2	161 (67.6)
e13/14a2	65 (27.3)
Undetermined/not tested/other	12 (5.0)

^a^ Including patients who did not report ethnicity and patients who reported their ethnicity as unknown

### Impact of clinical response on change in HRQoL

Least square (LS) mean changes from baseline were significantly better (*p* < 0.05) when assessed at the time of achieving CR/CRi vs. not achieving CR/CRi across all PRO domains of interest (Table 2, Figure S1). Among these domains, the differences in LS mean changes were considered clinically meaningful (i.e., ≥ published MID thresholds) for the FACT-Leu TOI (5.28 [1.99, 8.57]; MID = ± 5), the FACT-G total score (3.82 [0.93, 6.71]; MID = ± 3), and the FACT-Leu total score (7.18 [2.99, 11.38]; MID = ± 6). The magnitude of the differences in LS mean changes did not significantly differ between treatment groups based on the interaction tests. Furthermore, LS mean changes from baseline were consistent regardless of MRD-negative status or different levels of BCR-ABL1/ABL1 (Table 2, Figure S1).

**Table 2:** Association of clinical response and patient-reported overall treatment tolerability with change in HRQoL over time

		β (95% CI)							
Variable	*N*	FACT-Leu PWB (MID = ± 2)	FACT-LeuS (MID = ± 4)	FACT-Leu TOI (MID = ± 5)	FACT-G Total Score (MID = ± 3)	FACT-Leu Total Score (MID = ± 6)	EQ-VAS (MID = ± 7)	EQ-5D-5 L UK-based HUI (MID = ± 0.08)	EQ-5D-5 L US-based HUI (MID = ± 0.08)
Clinical response									
CR/CRi vs. No CR/CRi	566 vs. 52	1.24 (0.38, 2.09)	3.26 (1.34, 5.18)	5.28 (1.99, 8.57)	3.82 (0.93, 6.71)	7.18 (2.99, 11.38)	4.60 (0.47, 8.73)	0.04 (0.00, 0.08)	0.02 (˗0.01, 0.05)
p-value		0.005^a^	< 0.001^b^	0.002^a^	0.010^c^	< 0.001^c^	0.029^d^	0.081^e^	0.182^e^
MRD- CR vs. no CR	334 vs. 52	1.15 (0.25, 2.05)	3.02 (0.98, 5.06)	4.92 (1.42, 8.42)	3.72 (0.69, 6.74)	6.75 (2.31, 11.19)	4.32 (˗0.06, 8.70)	0.02 (˗0.02, 0.07)	0.01 (˗0.02, 0.04)
MRD + CR vs. no CR	215 vs. 52	1.41 (0.49, 2.33)	3.54 (1.46, 5.62)	5.87 (2.31, 9.43)	4.17 (1.07, 7.28)	8.06 (3.50, 12.63)	4.65 (0.11, 9.20)	0.05 (0.01, 0.10)	0.03 (˗0.01, 0.06)
p-value		0.011^a^	0.003^b^	0.005^a^	0.026^c^	0.002^f^	0.110^d^	0.047^b^	0.181^e^
BCR-ABL1/ABL1 ratio ≤ 0.01% CR vs. no CR	334 vs. 52	1.16 (0.26, 2.07)	3.08 (1.03, 5.13)	4.97 (1.45, 8.49)	3.58 (0.47, 6.69)	6.76 (2.29, 11.23)	4.40 (0.0, 8.79)	0.02 (˗0.02, 0.06)	0.01 (˗0.02, 0.05)
BCR-ABL1/ABL1 ratio > 0.01%−0.1% CR vs. no CR	111 vs. 52	1.47 (0.46, 2.47)	3.74 (1.47, 6.01)	6.01 (2.13, 9.89)	3.99 (0.55, 7.42)	8.03 (3.04, 13.02)	5.38 (0.45, 10.30)	0.04 (˗0.01, 0.09)	0.03 (˗0.01, 0.06)
BCR-ABL1/ABL1 ratio > 0.1%−1% CR vs. no CR	83 vs. 52	1.40 (0.35, 2.44)	3.57 (1.22, 5.93)	5.93 (1.89, 9.96)	4.01 (0.45, 7.58)	8.23 (3.05, 13.42)	3.60 (˗1.64, 8.84)	0.07 (0.02, 0.12)	0.04 (0.01, 0.08)
BCR-ABL1/ABL1 ratio > 1% CR vs. no CR	21 vs. 52	1.20 (˗0.22, 2.63)	2.68 (˗0.49, 5.85)	5.17 (˗0.26, 10.59)	3.92 (˗0.85, 8.70)	7.74 (0.82, 14.66)	4.45 (˗2.66, 11.56)	0.03 (˗0.04, 0.10)	0.01 (˗0.06, 0.04)
p-value		0.057^a^	0.019^b^	0.031^a^	0.165^c^	0.016^f^	0.281^d^	0.055^b^	0.107^e^
FACT-GP5 (‘I am bothered by side effects of treatment’) ^g^									
“A little bit” vs. “Not at all”	278 vs. 192	˗2.53 (˗3.09, ˗1.96)	˗2.68 (˗3.94, ˗1.42)	˗6.16 (˗8.32, ˗4.01)	˗4.01 (˗5.90, ˗2.12)	˗6.26 (˗9.02, ˗3.51)	˗3.85 (˗6.64, ˗1.07)	˗0.07 (˗0.09, ˗0.04)	˗0.05 (˗0.07, ˗0.04)
“Somewhat” vs. “Not at all”	105 vs. 192	˗5.50 (˗6.24, ˗4.76)	˗5.67 (˗7.34, ˗4.00)	˗12.75 (˗15.60, ˗9.89)	˗9.04 (˗11.54, ˗6.55)	˗14.11 (˗17.77, ˗10.46)	˗9.14 (˗12.78, ˗5.50)	˗0.09 (˗0.12, ˗0.05)	˗0.06 (˗0.09, ˗0.04)
“Quite a bit”/”Very much” vs. “Not at all” ^h^	53 vs. 192	˗9.90 (˗10.84, ˗8.96)	˗10.51 (˗12.65, ˗8.37)	˗23.28 (˗26.92, ˗19.63)	˗16.82 (˗20.00, ˗13.64)	˗26.82 (˗31.50, ˗22.14)	˗15.09 (˗19.76, ˗10.42)	˗0.24 (˗0.29, ˗0.20)	˗0.18 (˗0.21, ˗0.14)
p-value		< 0.001^a^	< 0.001^b^	< 0.001^a^	< 0.001^c^	< 0.001^c^	< 0.001^d^	< 0.001^e^	< 0.001^e^

Prespecified MID thresholds were used to determine whether the differences were clinically meaningful [25–30]. N represents the numbers of observations within each subgroup for FACT-Leu PWB assessments. As this is a repeated measure analysis, a patient may contribute data at multiple time points. The numbers of observations for other primary PRO domains were similar

^a^ Adjusted for baseline score and sex. ^b^ Adjusted for baseline score, sex, and time from initial diagnosis of Ph + ALL to first dose date of prior anti-cancer regimen. ^c^ Adjusted for baseline score. ^d^ Adjusted for baseline score and age (years). ^e^ Adjusted for baseline score, age (years), sex, and time from initial diagnosis of Ph + ALL to first dose date of prior anti-cancer regimen. ^f^ Adjusted for baseline score and time from initial diagnosis of Ph + ALL to first dose of prior anti-cancer regimen. ^g^ FACT-GP5 was included as a predictor in the final model with the predictor of CR/CRi vs. no CR/CRi. ^h^ The FACT-G item GP5 categories “Quite a bit” and “Very much” were combined due to the small sample size for each response level

Abbreviations: ALL, acute lymphoblastic leukemia; BCR-ABL, breakpoint cluster region-Abelson; CI, confidence interval; CR, complete remission; FACT-G, functional assessment of cancer therapy-general; FACT-Leu, functional assessment of cancer therapy-leukemia; HUI, health utility index; HRQoL, health-related quality of life; LeuS, leukemia “additional concerns” subscale; MID, minimal important difference; MRD, minimal residual disease; Ph+, Philadelphia positive; TOI, trial outcome index; UK, United Kingdom; US, United States; VAS, visual analogue scale [17, 25–30]

### Impact of patient-reported overall treatment tolerability on change in HRQoL

The proportion of patients who reported being “not at all” bothered by side effects of treatment was consistently higher in the ponatinib arm than in the imatinib arm at C4D1, C7D1, and the end of treatment (Table S4). Patient-reported treatment intolerability, as assessed by the FACT-GP5, was associated with significant and clinically meaningful worsening in almost all primary PRO domains of interest (Table 2). The level of worsening in HRQoL substantially increased with a greater degree of bother by treatment side effects. This trend was clear and consistent across all PRO domains of interest (Table 2, Figure S1). The interaction test did not find that associations between overall treatment intolerability and change in HRQoL significantly differed by treatment group.

## Discussion

This study provides deeper insights into the benefits of ponatinib over imatinib in HRQoL by examining the relationships between clinical response, patient-reported overall treatment tolerability, and HRQoL based on data from the phase 3 PhALLCON trial. Previous analyses of PRO data from the PhALLCON study demonstrated treatment benefits of ponatinib over imatinib across multiple PRO endpoints [13]. This analysis aimed to further understand and contextualize these PRO findings. Findings from the current study indicate that change in HRQoL from baseline across all domains of interest was significantly better when CR/CRi was achieved after treatment with TKI (ponatinib or imatinib, plus reduced-intensity chemotherapy). Further, the change in HRQoL was considered clinically meaningful for most PRO domains. However, a deeper CR at molecular level did not result in a greater improvement in HRQoL, based on the domains examined in this study. Patient-reported overall treatment tolerability was associated with significant and clinically meaningful worsening of HRQoL across all domains, with a higher degree of “bother” due to side effects from TKI therapy associated with a greater level of worsening in HRQoL.

Few studies have examined the impact of clinical response on HRQoL for patients treated with TKIs. Yu et al. showed that effective TKI treatment (MR within 12 months after treatment with imatinib or nilotinib) was associated with improvement in physical and mental health in patients with chronic myeloid leukemia [34]. An analysis of pooled data from the bosutinib and imatinib arms in the phase 3 BFORE trial showed that achieving a deep MR was associated with improvements in the FACT-G and FACT-Leu domain scores in adults with newly diagnosed chronic phase chronic myeloid leukemia [26]. The current analysis demonstrated that achieving clinical response was associated with significantly better changes from baseline in all primary PRO domains, indicating that patients experienced better well-being and functional status and reduced symptoms when responding to TKI therapy. Given that the ponatinib arm achieved a very high overall response rate (93.9% of CR or CRi by the end of induction and 95.1% by the end of consolidation phase), and much longer duration of CR than imatinib (not reached for ponatinib vs. 22.3 months) [6], the findings from the current analysis provide further evidence for the positive impact of ponatinib on HRQoL over imatinib that was previously reported [13].

The depth of MRD-negative CR has been correlated with improved outcomes for patients with Ph + ALL [35, 36]. A study in adults with ALL found positive impact of MRD-negative status, compared to MRD-positive status, on HRQoL, without considering the achievement of CR [37]. In the current study, achieving clinical response (CR or CRi), regardless of MRD-negative status or different levels of BCR-ABL1/ABL1, resulted in a greater improvement in HRQoL, as compared to no clinical response. The results from the BFORE trial showed that patients who achieved a deeper molecular response experienced significantly greater improvements in emotional well-being and leukemia-specific domains [26]. In contrast, our analysis did not find that deeper molecular response led to greater improvements in HRQoL. This discrepancy may be explained by the following two reasons. First, the definition of MRD-negative status differed between the two analyses: the achievement of CR was not considered in their definition, whereas it was included in our definition. Secondly, our models controlled for potential confounders such as baseline score, age, sex, and time from initial diagnosis to the first dose of prior anti-cancer therapy. In contrast, the BFORE study did not adjust for potential confounding factors. These differences may help to explain the discrepancy in findings between the two studies. Our study found that achieving deeper molecular response beyond complete remission (CR) did not results in additional improvement in HRQoL. This finding suggests that HRQoL may be more strongly influenced by the presence or resolution of disease-related symptoms, typically addressed by CR rather than by the depth of molecular response, which may not correlate with symptomatic burden. In the current study, the degree to which patients were bothered by treatment-related side effects was associated with significant and clinically meaningful changes in scores across all PRO domains of interest. Higher degree of “being bothered” by side effects of treatment was associated with greater worsening of HRQoL across all PRO domains. These findings further validate the benefit of ponatinib, compared to imatinib, in improving patients’ HRQoL by improving overall treatment tolerability. Further, the overall treatment tolerability appears to have a much greater impact than clinical response on patients’ HRQoL. This conclusion is supported by a much larger magnitude of changes in PRO scores associated with the degree of “being bothered” by treatment side effects, compared to that associated with the level of clinical response. These results highlight the need to consider potential treatment toxicity alongside clinical effectiveness when selecting a TKI for patients, and the importance of close monitoring and timely management of side effects when they occur.

Although ponatinib has historically been associated with significant toxicities, particularly in older adults [38–40], findings from PhALLCON contrast with this perception, demonstrating better overall tolerability compared to imatinib. This shift is supported by emerging evidence that modern treatment strategies such as response-based dosing can enhance ponatinib’s overall tolerability [9, 41–45]. For example, a recent analysis by Jabour et al. comparing patients in the PACE and OPTIC trials showed that a response-guided dose-reduction strategy led to fewer adverse event–related dose reductions, longer treatment durations, and preserved efficacy [46]. While these findings are encouraging, further validation in real-world settings is needed to characterize the tolerability and quality-of-life impact of ponatinib across diverse patient populations.

A few limitations should be noted. Selection bias is possible in this study, since most patients who failed to achieve MRD-negative CR at the end of the induction phase discontinued study treatment. As PRO data were captured only once at the end of treatment visit, PRO data from those who had a suboptimal response to treatment and/or discontinued treatment due to unacceptable treatment toxicity were not well represented in the analysis. Therefore, the magnitude of the impact of achieving clinical response on the patients’ HRQoL may have been underestimated. Additionally, treatment with TKIs is associated with adverse events and side effects such as fatigue, nausea, vascular toxicity, hypertension, atrial fibrillation, reduced cardiac function, and heart failure [41, 46–49]. As the primary objective of this study was to explore the relationship between patient-reported “overall” treatment tolerability and changes in HRQoL, physician-reported adverse events were not included in the analysis. However, if the study objective were to examine the impact of “specific” treatment-related adverse event (e.g., fatigue, pain, nausea, vomiting) on HRQoL and/or patient-reported tolerability, incorporating physician-reported adverse event data would be necessary. Finally, while the results support an association between CR or MRD-negative CR and PRO benefits in patients with Ph + ALL, they do not provide formal validation of these measures as surrogate endpoints.

To our knowledge, this is the first study investigating the association of clinical response and patient-reported overall treatment tolerability with change in HRQoL in patients with Ph + ALL. Achieving clinical response after TKI therapy was linked to significantly better HRQoL, while treatment-related side effects led to significantly and clinically meaningfully worse HRQoL. The influence of overall treatment tolerability on HRQoL appears to be greater than that of clinical response. Findings from this study support the clinical utility of clinical responses, such as CR or MRD-negative CR, in reasonably predicting HRQoL, although formal statistical validation of these surrogate endpoints is still needed. Our findings further reinforce the PRO benefits of ponatinib versus imatinib observed in the PhALLCON trial. These insights further support the use of ponatinib as a frontline treatment for Ph + ALL and provide timely information for clinicians and patients to guide them in treatment-related decision-making.

## Supplementary Information

Below is the link to the electronic supplementary material.

Supplementary Material 1 (DOCX. 222 KB)

## Data Availability

Data sets including the redacted study protocol, redacted statistical analysis plan, and individual participant data will be made available from the completed study, within three months from initial request to researchers whose proposals meet research criteria and other conditions. Data will be provided after de-identification, in compliance with applicable privacy laws, data protection, and requirements for consent and anonymization.

## Abbreviations

BCR-ABL: breakpoint cluster region-Abelson
ECOG: Eastern Cooperative Oncology Group
SD: standard deviation

## Declarations

### Data sharing

Data sets including the redacted study protocol, redacted statistical analysis plan, and individual participant data will be made available from the completed study, within three months from initial request to researchers whose proposals meet research criteria and other conditions. Data will be provided after de-identification, in compliance with applicable privacy laws, data protection, and requirements for consent and anonymization.
