# Supplementary material for: Impact of clinical response and treatment tolerability on HRQoL in newly diagnosed Philadelphia chromosome-positive acute lymphoblastic leukemia patients treated with ponatinib or imatinib
Source: Ann Hematol. 2025 Oct 10;104(9):4669–78. doi: 10.1007/s00277-025-06635-0 (PMC12552232; doi:10.1007/s00277-025-06635-0)
Supplement: Supplementary file 1 — Supplementary Material 1 (DOCX. 222 KB) [file 277_2025_6635_MOESM1_ESM.docx]

**Impact of clinical response and treatment tolerability on HRQoL in newly diagnosed Philadelphia chromosome-positive acute lymphoblastic leukemia patients treated with ponatinib or imatinib**

Ajibade Ashaye MD MBA MPH MSc,^1^ Ling Shi PhD,^2^ Ibrahim Aldoss MD,^3^ Pau Montesinos MD PhD,^4^ Pankit Vachhani MD,^5^ Vanderson Rocha MD PhD MS,^6^ Cristina Papayannidis MD PhD,^7^ Jessica T. Leonard MD,^8^ Maria R. Baer MD,^9^ Jose-Maria Ribera MD PhD,^10^ Yanyu Wu MSc PhD,^1^ Meliessa Hennessy MPH,^1^ Alexandar Vorog MD,^1^ Shien Guo PhD^2^

^1^Takeda Development Center Americas, Inc., Cambridge, MA, USA

^2^Evidera, PPD clinical research business of Thermo Fisher Scientific, Waltham, MA, USA

^3^City of Hope National Medical Center, Duarte, CA, USA

^4^Hospital Universitari i Politècnic La Fe, Valencia, Spain

^5^University of Alabama at Birmingham, Birmingham, AL, USA

^6^Instituto do Câncer do Estado de São Paulo, University of São Paulo, São Paulo, Brazil

^7^IRCCS Azienda Ospedaliero-Universitaria di Bologna, Istituto di Ematologia “L. e A. Seràgnoli,” Bologna, Italy

^8^Oregon Health and Science University, Portland, OR, USA

^9^University of Maryland Marlene and Stewart Greenebaum Comprehensive Cancer Center, Baltimore, MD, USA

^10^ICO – Hospital Germans Trias i Pujol, Josep Carreras Leukaemia Research Institute, Badalona, Spain

**Corresponding author details:**

Ajibade Ashaye MD MBA

Takeda Development Center Americas, Inc.

Cambridge, MA, USA

[ajibade.ashaye@takeda.com](mailto:ajibade.ashaye@takeda.com)

Phone: 617-679-7000

# Supplemental Appendix

## Table S1. FACT-Leu score ranges

| **Domain/subscale** | **Number of items** | **Items** | **Score range** [1] |
| --- | --- | --- | --- |
| FACT-G PWB | 7 | GP1, GP2, GP3, GP4, GP5, GP6, GP7 | 0–28 |
| FACT-G SWB | 7 | GS1, GS2, GS3, GS4, GS5, GS6, GS7 | 0–28 |
| FACT-G EWB | 6 | GE1, GE2, GE3, GE4, GE5, GE6 | 0–24 |
| FACT-G FWB | 7 | GF1, GF2, GF3, GF4, GF5, GF6, GF7 | 0–28 |
| FACT-LeuS | 17 | BRM3, P2, BRM2, ES3, LEU1, TH1, TH2, HI12, BMT6, C2, C6, An7, N3, LEU5, LEU6, BRM9, LEU7 | 0–68 |
| FACT-Leu TOI | 31 | PWB + FWB + FACT-LeuS | 0–124 |
| FACT-G total score | 27 | PWB + SWB + EWB +FWB | 0–108 |
| FACT-Leu total score | 44 | FACT-G + FACT-LeuS | 0–124 |

Abbreviations: BRM, biologic response modifier; EWB, emotional well-being; FACT-G, functional assessment of cancer therapy-general; FACT-Leu, functional assessment of cancer therapy-leukemia; FWB, functional well-being; GP, general population; HRQoL, health-related quality of life; Leu, leukemia; LeuS, leukemia “additional concerns” subscale; PWB, physical well-being; SWB, social/family well-being; TOI, trial outcome index.

Items for which a higher value indicated worse HRQoL were reverse scored so that a higher score indicated better HRQoL [1]. Domain scores were calculated only if >50% of the items were non-missing [1]. FACT-G total score, FACT-Leu total score, and FACT-Leu TOI were calculated only if >80% of all items were non-missing [1].

## Table S2. Potential Determinants of HRQoL Examined in the MMRM models

| **Determinant** | **Levels** |
| --- | --- |
| **Baseline Factors (Time-independent)** | |
| Age (years) | Continuous |
| Age group | <45, 45 to <60, ≥60 years |
| Sex | Male, female |
| Race | White, non-White |
| Ethnicity | Hispanic or Latino, Not Hispanic or Latino |
| ECOG performance status | 0, 1, 2 |
| Baseline BCR-ABL1 transcript type | P190, P210 |
| Time from initial diagnosis of Ph+ ALL to first dose of previous anti-cancer regimen (days) | Continuous |
| Extramedullary disease | Yes/No |
| **Time-varying Factors** | |
| Clinical response^a^ | CR/CRi vs no CR/CRi |
|  | MRD-negative CR vs. MRD-positive CR vs. no CR |
|  | BCR-ABL1/ABL1 ratio ≤0.01% CR |
|  | BCR-ABL1/ABL1 ratio >0.01%–0.1% CR |
|  | BCR-ABL1/ABL1 ratio >0.1%–1% CR |
|  | BCR-ABL1/ABL1 ratio >1%–10% CR |
|  | BCR-ABL1/ABL1 ratio >10% CR |
|  | No CR |
| Patient-reported overall treatment tolerability | FACT-G item GP5 (“not at all”, “a little bit”, “somewhat”, “quite a bit”, “very much") |

Abbreviations: ALL, acute lymphoblastic leukemia; BCR-ABL1, breakpoint cluster region-Abelson proto-oncogene 1; CR, complete remission; ECOG, eastern cooperative oncology group; FACT-G, functional assessment of cancer therapy-general; HRQoL, health-related quality of life; MMRM, mixed-effects regression models for repeated-measures; MRD, minimal residual disease; Ph+, Philadelphia chromosome-positive.

^a^Each of the clinical response variables was examined in a separate model along with patient-reported overall treatment tolerability.

## Table S3. Summary of PRO completion by CR/CRi status

| **PRO Visit** | **Number of patients  with PRO data** | **Number of patients achieving CR/CRi** | **Number of patients without achieving CR/CRi** |
| --- | --- | --- | --- |
| Cycle 4 Day 1 | 194 | 177 | 17 |
| Cycle 7 Day 1 | 147 | 138 | 9 |
| Cycle 10 Day 1 | 84 | 73 | 11 |
| Cycle 13 Day 1 | 57 | 52 | 5 |
| Cycle 16 Day 1 | 47 | 43 | 4 |
| Cycle 19 Day 1 | 41 | 41 | 0 |
| Cycle 21 Day 1 | 35 | 30 | 5 |

Abbreviations: CR, complete remission; CRi, CR with incomplete hematologic recovery; PRO, patient-reported outcome.

## Table S4. Participants’ responses to the FACT-GP5

| **Visit^a^** | **Ponatinib**  **n (%)** | **Imatinib**  **n (%)** | **p-value^b^** |
| --- | --- | --- | --- |
| Baseline (N) | 149 | 74 | 0.951 |
| Not at all | 92 (61.7) | 46 (62.2) |  |
| A little bit | 40 (26.8) | 20 (27.0) |  |
| Somewhat | 13 (8.7) | 6 (8.1) |  |
| Quite a bit. | 4 (2.7) | 2 (2.7) |  |
|  |  |  |  |
| C4D1 (N) | 122 | 55 | 0.053 |
| Not at all | 34 (27.9) | 8 (14.5) |  |
| A little bit | 54 (44.3) | 29 (52.7) |  |
| Somewhat | 22 (18.0) | 10 (18.2) |  |
| Quite a bit | 9 (7.4) | 4 (7.3) |  |
| Very much | 3 (2.5) | 4 (7.3) |  |
|  |  |  |  |
| C7D1 (N) | 77 | 23 | 0.446 |
| Not at all | 23 (29.9) | 5 (21.7) |  |
| A little bit | 36 (46.8) | 8 (34.8) |  |
| Somewhat | 13 (16.9) | 9 (39.1) |  |
| Quite a bit | 3 (3.9) | 0 (0.0) |  |
| Very much | 2 (2.6) | 1 (4.3) |  |
|  |  |  |  |
| EOT (N) | 69 | 51 | 0.017 |
| Not at all | 26 (37.7) | 9 (17.6) |  |
| A little bit | 24 (34.8) | 19 (37.3) |  |
| Somewhat | 12 (17.4) | 15 (29.4) |  |
| Quite a bit | 6 (8.7) | 5 (9.8) |  |
| Very much | 1 (1.4) | 3 (5.9) |  |

Abbreviations: C, cycle; D, day; EOT, end of treatment; FACT-G, functional assessment of cancer therapy-general.

^a^ Reported up to the visit with n≥ 20 in each arm.

^b^ p-value from Chi-square test based on the proportion of participants who responded “Not at all” to the FACT-Item GP5 (‘I am bothered by side effects of treatment’).

## Figure S1 Forest plot of LS mean change from baseline

Panel A presents the difference in LS change from baseline in HRQoL between achieving clinical response versus not achieving clinical response. Clinical response was defined based on investigator-reported CR/CRi and depth of molecular response levels. Panel B presents the difference in LS change from baseline in HRQoL by levels of the FACT-GP5 ‘bothered by treatment side effects”, with “Not at all” as the reference group. FACT-GP5 was included as a predictor in the final model with the predictor of CR/CRi vs no CR/CRi.

Prespecified MID thresholds were used to determine whether the differences were clinically meaningful [2-7]. ^a^ Treatment tolerability was assessed by the patient-reported FACT-Item GP5 (‘I am bothered by side effects of treatment’), with response levels on a 5-point scale ranging from 0 (“Not at all”) to 4 (“Very much”) [8]. The FACT-Item GP5 categories “Quite a bit” and “Very much” were combined due to the small sample size for each response level.  ^b^ Adjusted for baseline score and sex. ^c^ Adjusted for baseline score, sex, and time from initial diagnosis of Ph+ ALL to first dose date of prior anti-cancer regimen. ^d^ Adjusted for baseline score. ^e^ Adjusted for baseline score and age (years). ^f^Adjusted for baseline score, age (years), sex, and time from initial diagnosis of Ph+ ALL to first dose date of prior anti-cancer regimen. ^g^ Adjusted for baseline score and time from initial diagnosis of Ph+ ALL to first dose of prior anti-cancer regimen.

Abbreviations: BCR-ABL, breakpoint cluster region-Abelson; CI, confidence interval; CR, complete remission; FACT-G, functional assessment of cancer therapy-general; FACT-Leu, functional assessment of cancer therapy-leukemia; HUI, health utility index; HRQoL, health-related quality of life; iCR, incomplete remission; LeuS, leukemia “additional concerns” subscale; LS, least square; MID, minimal important difference; MRD, minimal residual disease; TOI, trial outcome index; UK, United Kingdom; US, United States; VAS, visual analogue scale.

# REFERENCES

1. FACIT Group (2021) Scoring. <https://www.facit.org/scoring>. Accessed August 31 2024

2. Webster K, Cella D, Yost K (2003) The Functional Assessment of Chronic Illness Therapy (FACIT) Measurement System: properties, applications, and interpretation. Health Qual Life Outcomes 1 (1):1-7. doi:10.1186/1477-7525-1-79

3. Brümmendorf TH, Gambacorti-Passerini C, Bushmakin AG, Cappelleri JC, Viqueira A, Reisman A, Isfort S, Mamolo C (2020) Relationship between molecular response and quality of life with bosutinib or imatinib for chronic myeloid leukemia. Ann Hematol 99 (6):1241-1249. doi:10.1007/s00277-020-04018-1

4. Trask PC, Cella D, Besson N, Kelly V, Masszi T, Kim DW (2012) Health-related quality of life of bosutinib (SKI-606) in imatinib-resistant or imatinib-intolerant chronic phase chronic myeloid leukemia. Leuk Res 36 (4):438-442. doi:10.1016/j.leukres.2011.10.011

5. Whiteley J, Reisman A, Shapiro M, Cortes J, Cella D (2016) Health-related quality of life during bosutinib (SKI-606) therapy in patients with advanced chronic myeloid leukemia after imatinib failure. Curr Med Res Opin 32 (8):1325-1334. doi:10.1185/03007995.2016.1174108

6. Cella D, Hahn EA, Dineen K (2002) Meaningful change in cancer-specific quality of life scores: differences between improvement and worsening. Qual Life Res 11 (3):207-221. doi:10.1023/a:1015276414526

7. Pickard AS, Neary MP, Cella D (2007) Estimation of minimally important differences in EQ-5D utility and VAS scores in cancer. Health Qual Life Outcomes 5:70

8. Pearman TP, Beaumont JL, Mroczek D, O'Connor M, Cella D (2018) Validity and usefulness of a single-item measure of patient-reported bother from side effects of cancer therapy. Cancer 124 (5):991-997. doi:10.1002/cncr.31133
